# Group A rotavirus surveillance before vaccine introduction in Italy, September 2014 to August 2017

**DOI:** 10.2807/1560-7917.ES.2019.24.15.1800418

**Published:** 2019-04-11

**Authors:** Giovanni Ianiro, Roberto Micolano, Ilaria Di Bartolo, Gaia Scavia, Marina Monini, RotaNet-Italy Study Group

**Affiliations:** 1 Department of Food Safety, Nutrition and Veterinary Public Health, Istituto Superiore di Sanità, Rome, Italy; 2 The members of the RotaNet-Italy Study Group who contributed data are listed at the end of this article

**Keywords:** group A rotavirus, genotype, acute gastroenteritis, human, Italy, epidemiology, infection control, molecular methods, rotavirus, rotavirus infection, statistics, surveillance, viral infections

## Abstract

**Introduction:**

Group A rotaviruses (RVA) are the leading cause of acute gastroenteritis (AGE) in young children, causing ca 250,000 deaths worldwide, mainly in low-income countries. Two proteins, VP7 (glycoprotein, G genotype) and VP4 (protease-sensitive protein, P genotype), are the basis for the binary RVA nomenclature. Although 36 G types and 51 P types are presently known, most RVA infections in humans worldwide are related to five G/P combinations: G1P[8], G2P[4], G3P[8], G4P[8], G9P[8].

**Aim:**

This study aimed to characterise the RVA strains circulating in Italy in the pre-vaccination era, to define the trends of circulation of genotypes in the Italian paediatric population.

**Methods:**

Between September 2014 and August 2017, after routine screening in hospital by commercial antigen detection kit, 2,202 rotavirus-positive samples were collected in Italy from children hospitalised with AGE; the viruses were genotyped following standard European protocols.

**Results:**

This 3-year study revealed an overall predominance of the G12P[8] genotype (544 of 2,202 cases; 24.70%), followed by G9P[8] (535/2,202; 24.30%), G1P[8] (459/2,202; 20.84%) and G4P[8] (371/2,202; 16.85%). G2P[4] and G3P[8] genotypes were detected at low rates (3.32% and 3.09%, respectively). Mixed infections accounted for 6.49% of cases (143/2,202), uncommon RVA strains for 0.41% of cases (9/2,202).

**Conclusions:**

The emergence of G12P[8] rotavirus in Italy, as in other countries, marks this genotype as the sixth most common human genotype. Continuous surveillance of RVA strains and monitoring of circulating genotypes are important for a better understanding of rotavirus evolution and genotype distribution, particularly regarding strains that may emerge from reassortment events.

## Introduction

Group A rotaviruses (RVA) are the leading cause of acute gastroenteritis (AGE) in young children worldwide, and are estimated to cause 250,000 deaths every year among children aged 0–5 years, mainly in low-income countries of sub-Saharan Africa and South East Asia [1].

Rotaviruses belong to the *Reoviridae* family, and possess a triple-layered icosahedral capsid enclosing a genome of 11 segments of double-stranded RNA encoding six structural and five or six non-structural proteins. The RVA capsid consists of three protein layers, with the outer layer made up of the VP7 (capsid) and VP4 (spike) proteins [2,3].

The middle-layer protein VP6 defines nine antigenically distinct groups (A–I) [2,4]. The outer capsid proteins VP7 and VP4 define the G and P genotypes, respectively [5,6]. Based on nucleotide differences in genes 9 (VP7) and 4 (VP4), RVA are currently classified in 36 G and 51 P genotypes [7]. Although several G/P genotype combinations have been reported [8,9], the RVA genotypes G1P[8], G2P[4], G3P[8], G4P[8] and G9P[8] have caused together up to three quarters of human RVA infections worldwide. The only exception is Africa, where a different epidemiology from the rest of the world has been observed, with a greater genotype diversity among RVA affecting humans [10].

RVA are recognised as the leading cause of AGE in the paediatric population (< 5 years-old), but older children and adults may also have symptomatic infections. However, the role of RVA infections in adult gastroenteritis has been poorly investigated [11]. The continuing evolution of RVA is linked to two typical mechanisms of viruses possessing a segmented genome: genetic drift and genetic shift. The former is caused by the accumulation of random point mutations owing to the error-prone nature of the viral RNA-dependent RNA polymerase. The latter can occur during infection of the same host cell with two different viral variants through the exchange of one or more complete genomic segments [9,12].

In 2006, two live-attenuated RVA vaccines were licensed for human use and have since then been introduced into national immunisation programmes in an increasing number of countries worldwide. Their antigenic compositions are based on the most common RVA genotypes circulating worldwide: the monovalent vaccine Rotarix, which is based on human G1 and P[8] antigen specificities, derived from a single human live attenuated G1P[8] RVA strain, and the pentavalent vaccine Rotateq, which is based on G1, G2, G3, G4 and P[8] antigens, derived from five live attenuated human-bovine reassortant RVA strains [13-15]. Both vaccines provide high protection against RVA AGE, resulting in a large decrease of the RVA AGE-related mortality (up to 53% reduction) and hospitalisations (up to 47% reduction) after the implementation of vaccination [16].

As part of the European Rotavirus Network (EuroRotaNet, http://www.eurorota.net/), an Italian national surveillance network (RotaNet-Italy study group) for RVA gastroenteritis has been active since 2007. The molecular surveillance performed in Italy under the framework of the EuroRotaNet project in the time period from 2007 to 2009 showed that RVA strains possessing the G1 to G4 and G9 G genotypes and the P[4] and P[8] P genotypes were the most common cause of RVA AGE [17], in agreement with previous studies conducted worldwide [8,18].

During winter 2017, anti-RVA vaccination was added to the list of recommended vaccines to be administered to the paediatric population in Italy. Both Rotarix and Rotateq are available on the market and their administration is not mandatory. In addition, a debate about the benefits of anti-RVA vaccination is still ongoing among physicians [19]. For these reasons, the anti-RVA vaccination coverage in Italy is still low (8%) and not homogeneous among the different Italian regions (range: 0–40%), which have autonomy in the decision about non-mandatory vaccinations and started the programmes at different times [20].

Since the first detection of the G12 strain L26 (G12P[4]) in the Philippines in 1987 [21], this unusual RVA genotype associated with different human VP4 P genotypes has been reported as the cause of AGE in human sporadic cases [22-24]. In the past few years, reports of G12 RVA associated with the P[8] VP4 genotype have increased remarkably in several countries, including Brazil, Mexico, Spain and the United States[25-28], so that the emerging G12P[8] is now considered one of the major human genotypes circulating worldwide. The G12P[8] RVA strains were also detected in Italy in 2012 and 2013 when they were responsible for an outbreak in limited geographical areas [29,30].

This work was performed with the aim to characterise the RVA strains circulating in Italy during three consecutive RVA surveillance seasons before the introduction of anti-RVA vaccination at national level. This paper reports the obtained genotyping results and describes the spread of the G12P[8] RVA strains in the Italian paediatric population. 

## Methods

### Study design

This study was conducted as passive surveillance in hospitals of the Italian National Sanitary System (SSN) coordinated by the Ministry of Health, and located in 13 of the 20 Italian regions. The SSN hospitals involved in the study provided free inpatient care to the estimated 5,000,000 paediatric population living in the 13 regions involved. The study included patients from whom stool analysis was requested. An epidemic season was defined as the period from 1 September of one year to 31 August of the following year. 

The RVA passive surveillance in Italy has been active since 2007 and has been described previously [17]. Briefly, children admitted with AGE are enrolled following informed consent. Detailed demographic and clinical data are recorded and stool samples collected. RVA routine screening is conducted according to each hospital’s care protocol. 

### Laboratory surveillance of rotavirus infections in Italy

As part of the European rotavirus surveillance network EuroRotaNet, the laboratory surveillance of rotavirus in Italy is carried out through RotaNet-Italy, a network of 13 regional laboratories located in the main Italian hospitals which passively report, on a voluntary basis, the occurrence of cases of RVA infection and send the patients’ clinical samples to the Italian Public health Agency (Istituto Superiore di Sanità (ISS)). All laboratories of the Italian network used high-quality commercial immunochromatographic assays for antigen detection. All faecal samples screened as RVA-positive were confirmed and genotyped with molecular methods; no false positives were observed during the study period.

The population under study consisted of patients with symptoms of acute gastroenteritis admitted to paediatric hospitals or units and included mainly children aged 0–15 years. Nonetheless, it also sporadically includes adults in much smaller number.

Based on clinician requests, patients’ stool specimens are generally screened by routine standard methods for the presence of common enteric bacterial pathogens by the local hospital laboratory. During the winter season, the test of rotavirus A is usually performed first, instead of the bacterial tests. Most of the tests used to detect rotavirus antigen also detected adenovirus, and less frequently norovirus. Patients whose samples test positive for RVA are enrolled in the RotaNet-Italy framework. RVA-positive samples are then sent to the central ISS laboratory for molecular analysis and genotyping.

### Sample collection

Between September 2014 and August 2017, stool specimens were collected from children and adults hospitalised for RVA-AGE throughout Italy, and were genotyped for both VP7 and VP4. As established by EuroRotaNet, a rotavirus surveillance season was defined as the 12-month period between September and August of the following calendar year. A total of 700 patients with complete RVA genotype assignment was determined as the minimum required sample size to be enrolled in the study each year, according to the network’s guidelines and the Italian population size. All cases passively reported by the participating laboratories were prospectively enrolled over the surveillance period, starting on 1 September of each year. Rotavirus infection was diagnosed at the local hospitals by commercial antigen detection methods. Epidemiological information was obtained from RotaNet-Italy questionnaires filled in by the hospital staff (Supplement S1). Informed consent was obtained from all individual participants included in the study.

### Nucleic acid extraction, group A rotaviruses genotyping and nucleotide sequencing

Rotavirus genotyping was performed at the ISS following EuroRotaNet protocols [31], and genotype was assigned on the basis of the molecular size of PCR amplicons [5,6,32]. In order to confirm genotyping results, the identified G12P[8] RVA strains were also subjected to nucleotide sequencing of VP7 and VP4 gene segments.

The RVA sequences obtained in this study were deposited in GenBank, under the following accession numbers: from KY688157 to KY688176 for VP7 and from KY688177 to KY688181 for VP4.

### Statistical analysis

Categorical variables were described using counts and percentages. Mean and standard deviation (SD) were used to describe numerical variables. The U-Mann–Whitney test was performed to compare the age of patients among the different RVA genotype groups. Statistical trend analyses were carried out to evaluate the significance of temporal variations in the RVA genotypes. The autoregressive integrated moving average (ARIMA) model was implemented using p ≤ 0.05 as the value to identify a statistical significant trend, beyond chance. The outcome evaluated was the number of cases, assuming that no substantial background demographic changes had occurred in the study period.

### Ethical statement

Data are anonymous and provided through EuroRotaNet and the Italian SSN as routine rotavirus strain surveillance; therefore, for this type of study, ethical content is not required. 

## Results

### Cases reported by genotype and geographical area

Between September 2014 and August 2017, across three consecutive surveillance seasons, a total of 2,202 RVA-positive samples (775 samples in 2014/15; 712 samples in 2015/16; 715 samples in 2016/17) from patients with AGE were collected throughout Italy and analysed by molecular methods, in order to assign the G/P genotypes. Sampling was performed in 13 Italian regions, taking into account the population size of the regions involved in the surveillance, grouped as three major geographical areas: those located in northern Italy (population size age 0–14 years: 2,760,000), in central Italy (population size age 0–14 years: 2,161,000) and in southern Italy (population size age 0–14 years: 1,670,000) [33]. Considering the whole surveillance period, RVA-positive samples with available information on genotype were mostly from northern Italy (n = 1,097; 49.81%), followed by central Italy (n = 691; 31.38%) and southern Italy (n = 414; 18.81%). This pattern of distribution was also observed for every single surveillance season. In 2014/15, 275 (35.48%) positive samples were from northern Italy, 287 (37.04%) from central Italy and 213 (27.48%) from southern Italy. In 2015/16, 497 (69.80%) RVA-positive stool samples were from northern Italy, 162 (22.76%) from central Italy and 53 (7.44%) from southern Italy. In 2016/17, 325 (45.45%) samples were from northern Italy, 242 (33.85%) from central Italy and 148 (20.70%) from southern Italy.

The Italian surveillance network is composed of 13 regions, covering almost all of the peninsular Italian population, with a theoretical sampling fraction per region of 7.69%. Only four of the 13 regions reached this value, while three of 13 regions were slightly below and four of 13 were considerably below the 7.69% value. 

Considering the whole study period, the G12P[8] genotype was detected at the highest rate in Italy (24.70% of all positive samples), followed by G9P[8] with 24.30%, G1P[8] with 20.84% and G4P[8] with 16.85%. The G2P[4] and G3P[8] genotypes were rarely detected (3.32% and 3.09%, respectively) (Table). Mixed RVA infections were present in 6.49% of samples, while uncommon genotypes (i.e. G9P[4], G10P[8] and G4P[6]) were detected in 0.41% of samples. In order to confirm the unexpectedly large number of G12 detected by genotyping, we sequenced the VP7 gene of 20 strains. In addition, five of these strains were randomly selected for nucleotide sequencing of the VP4 gene. All sequences confirmed the genotypes assigned by PCR for both VP7 and VP4 genes.

**Table:** Rotavirus genotypes circulating in Italy, September 2014–August 2017 (n = 2,202)

RVA genotypes	Italian geographical areas						Whole RotaNet-Italy area	
	Northern Italy		Central Italy		Southern Italy			
	n	%	n	%	n	%	n	%
G1P[8]	251	22.88	137	19.83	71	17.15	459	20.84
G2P[4]	17	1.56	31	4.49	25	6.04	73	3.32
G3P[8]	21	1.91	30	4.34	17	4.11	68	3.09
G4P[8]	130	11.85	116	16.79	125	30.19	371	16.85
G9P[8]	305	27.80	147	21.27	83	20.05	535	24.30
G12P[8]	295	26.89	187	27.06	62	14.98	544	24.70
Mixed	78	7.11	36	5.21	29	7.00	143	6.49
Uncommon	0	0	7	1.01	2	0.48	9	0.41
**Total**	**1,097**	**100**	**691**	**100**	**414**	**100**	**2,202**	**100**

The table reports numbers and percentages related to the RVA genotypes detected in all positive samples analysed in this study. The numbers cover the whole RotaNet-Italy area (n = 2,202) and the involved regions are divided in three major Italian geographical areas: northern Italy (n = 1,097), central Italy (n = 691) and southern Italy, including Sardinia island (n = 414). The 100% values refer to the total number of RVA genotypes detected for each geographical area.

**Figure 1:**
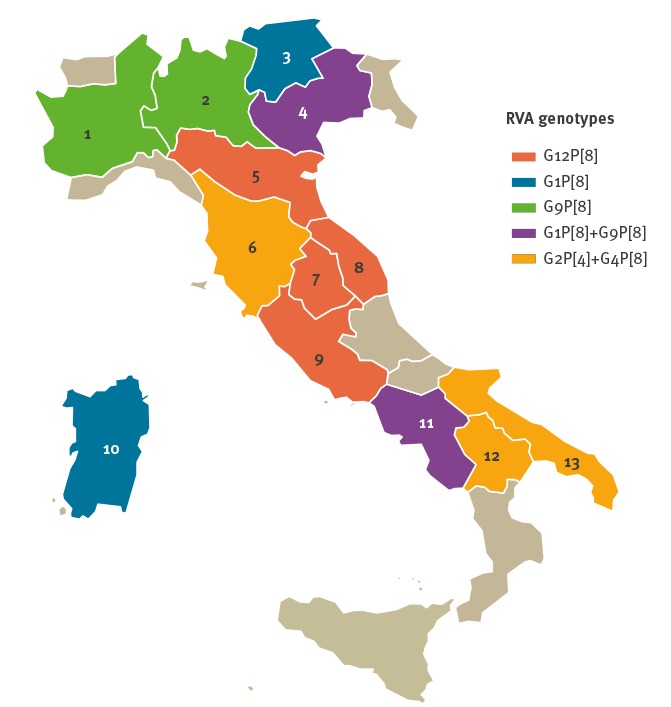
Geographical distribution of rotavirus genotypes circulating in Italy, September 2014–August 2017 (n = 2,202)

The analysis of RVA genotype distribution by geographical areas (northern, central and southern Italy) revealed important differences (Figure 1). While genotypes G1P[8] and G9P[8] were reported in similar proportions in the three investigated areas, the G12P[8] genotype was mainly identified in patients from northern and central Italy, whereas genotype G4P[8] was mostly reported in southern Italy. Stratified analysis by region showed that the G1P[8] genotype was the prevailing RVA genotype in patients from north-eastern regions of the country (Trentino, Veneto) and Sardinia, the G9P[8] in the whole of northern Italy (Lombardy, Piedmont, Veneto), the G12P[8] in central Italy (Emilia-Romagna, Umbria, Marche, Lazio), while the G4P[8] and G2P[4] genotypes was dominant in central (Tuscany) and southern Italy (Basilicata, Apulia).

RVA: group A rotavirus.

The different genotypes are colour-coded, highlighting the predominance for each Italian region involved in the surveillance. Regions correspond to the following numbers: 1 = Piedmont, 2 = Lombardy, 3 = Trentino-Alto Adige, 4 = Veneto, 5 = Emilia-Romagna, 6 = Tuscany, 7 = Umbria, 8 = Marche, 9 = Lazio, 10 = Sardinia, 11 = Campania, 12 = Basilicata and 13 = Apulia.

### Trends of circulation of rotavirus genotypes

**Figure 2:**
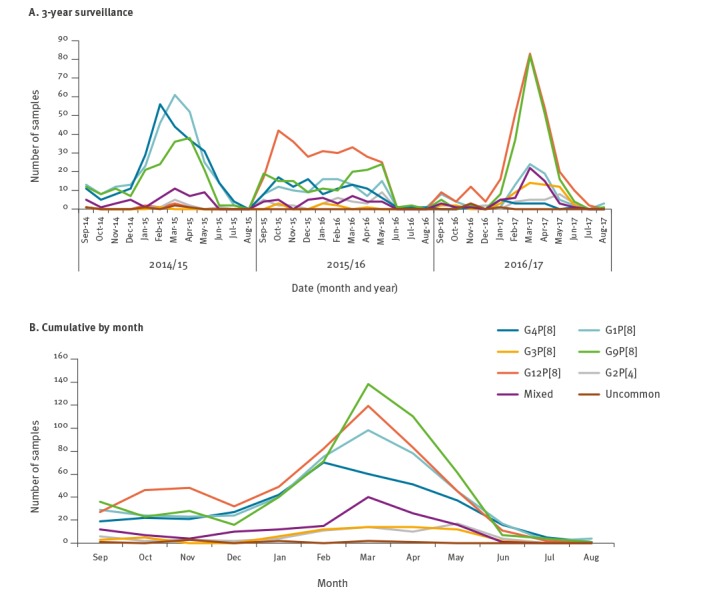
Temporal distribution of circulating rotavirus genotypes in Italy, September 2014–August 2017 (n = 2,202)

The distribution of RVA genotypes in the three investigated seasons is shown in Figure 2A. Yearly variations in the predominance of RVA genotypes were tested for statistical significance using the ARIMA model. Interestingly, the G12P[8] genotype was absent in the 2014/15 season, emerged and circulated in both the 2015/6 and 2016/7 season and became the most frequently reported RVA genotype across the country. Conversely, genotypes G1P[8] and G4P[8] peaked in the season 2014/15 and decreased progressively in the following two seasons. In genotype G1P[8], we observed a statistically significant decreasing trend of detection in the surveillance period, both for the whole study region (p = 0.041) and in northern Italy (p = 0.009). Similarly, decreasing trend for genotype G4P[8] was statistically significant both for the whole study region (p = 0.0007) and in central Italy (p = 0.0001). Finally, genotype G9P[8] circulated continuously during the whole period. The analysis of the temporal distribution did not yield other statistically significant results for the rest of RVA genotypes; this indicates that in the period from 2014 to 2017, the trends were stable or, alternatively, that any increase or decrease was too limited to exclude that variations were due to chance.

Total number of rotaviruses per season in Panel A: 2014/15: n = 775; 2015/16: n = 712; 2016/17: n = 715. Cumulative data for the three seasons by month are shown in Panel B.

### Seasonality

We analysed the seasonal distribution of the RVA genotypes circulating in Italy by considering the cumulative sum of detections during the entire study period per month (Figure 2B). When grouping the seasons, the G12P[8], G1P[8] and G9P[8] RVA genotypes showed a peak in March, while the G3P[8] genotype revealed a longer peak stretching across March and April. The G4P[8] genotype showed an early peak in February, and the G2P[4] a late peak in May (Figure 2B).

**Figure 3:**
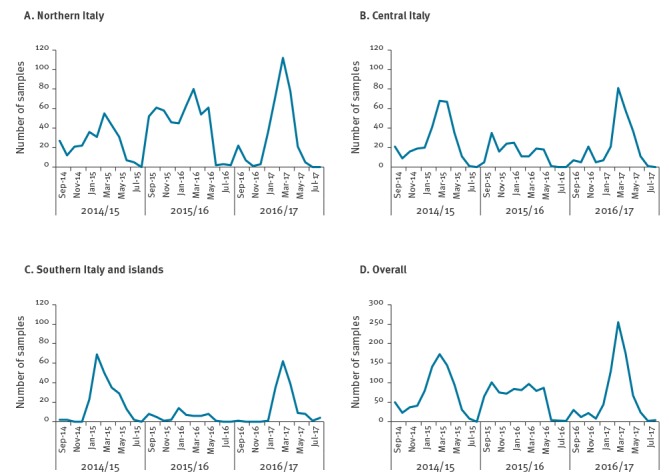
Passive and voluntary surveillance sampling of rotavirus in Italy, from patients hospitalised with acute gastroenteritis symptoms, September 2014–August 2017 (n = 2,202)

The circulation in the 3 years, both by genotype (Figure 2) and overall (Figure 3), revealed an absence of peaks in the middle of the 2015/16 season. The G12P[8] genotype increased and peaked for the first time in October 2015, with the highest number of G12-related AGE cases in March 2017, while the G1P[8] and G4P[8] genotypes peaked in March 2015 and February 2015, respectively, before a decrease in the following seasons. The G9P[8] showed high rates of detection across the three years, peaking always in March (Figure 2).

Sampling is shown for the three geographical areas of Italy and for the overall country. Northern Italy: n = 1,097, central Italy: n = 691, southern Italy: n = 414 and overall sampling: n = 2,202.

Despite the overall absence of a defined peak of the RVA infections during the 2015/16 season and the marked presence of peaks in 2014/15 and 2016/17, the RVA circulation in the three major geographical areas differed from the overall circulation (Figure 3). In northern Italy, the RVA peak was present in all three consecutive seasons. In detail, the 2015/16 peak was clearly visible, probably related to the oversampling in this area during the middle season, where samples from northern Italy represented 69.80% (497/712) of the total sampling. In central Italy, the distribution was similar to the overall RVA detection, with a flat and long distribution, while in southern Italy, only the 2014/15 and 2016/17 peaks were present and RVA samples were almost absent in 2015/16 (Figure 3).

### Demographic data

Considering the whole sample collection, the male/female ratio of RVA AGE-affected patients was 1.34 (for 16 cases the information was not available). Sex distribution by genotype showed that male cases predominated in all genotype groups. None of the patients enrolled in the surveillance had received anti-rotavirus vaccination before hospitalisation.

**Figure 4:**
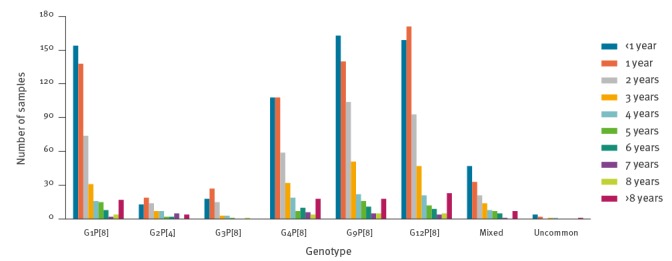
Rotavirus infections in Italy, by genotypes and age group, September 2014–August 2017 (n = 2,202)

The overall distribution of infections in different age groups revealed that most RVA infections occurred in children aged 0–24 months (1,310/2,202 cases; 59.49%) and 25–60 months (660/2,202; 29.97%). The median age of infected patients was 19.00 months (interquartile range (IQR): 24.00). Only 31 of 2,202 infections (1.41%) occurred in adult patients between 19 and 90 years of age. However, this information is expected to be strongly biased by the selective approach to the RVA surveillance scheme, which mainly included paediatric units. The G1P[8] was the only genotype infecting patients in all the age classes considered (Figure 4).

The total number of infections for each genotype are: G1P[8]: n = 459, G2P[4]: n = 73, G3P[8]: n = 68, G4P[8]: n = 371, G9P[8]: n = 535, G12P[8]: n = 544, mixed infections: n = 143 and uncommon genotypes: n = 9.

The statistical analysis revealed that patients affected by the G2P[4] genotype were significantly older (median age: 47.00 months; IQR: 35.00) than those affected by the G3P[8] (median age: 21.50 months; IQR: 15.25, difference: 25.50 months; p = 0.011), G4P[8] (median age: 31.90 months; IQR: 16.10, difference: 15.10 months; p = 0.020) and G9P[8] (median age: 29.75 months; IQR: 27.25, difference: 17.25 months; p = 0.005) genotypes. Moreover, the comparison between a single group of patients affected by a defined genotype and the rest of patients affected by all the other genotype combinations, revealed that the G12P[8] RVA genotype affected children significantly older (median age: 39.98 months; IQR: 23.00) than the other genotypes (p = 0.045) (Supplement S2).

## Discussion

This study reports the national surveillance of RVA infections during 36 months (September 2014 to August 2017) in Italy, showing for the first time since EuroRotaNet was established in 2007 that RVA genotype G12P[8] predominated over the five most common genotypes circulating worldwide (G1P[8], G2P[4], G3P[8], G4P[8] and G9P[8]) [17]. Since 2015, the G12P[8] genotype has been reported in Italy [29,30]. Also other findings indicate that this genotype can be considered an emerging RVA genotype and adapted to the human host [34].

Our genotyping results collected during three consecutive RVA surveillance seasons revealed that in the 2014/15 season, the RVA genotypes circulation was the same as in all seasons since 2007, with a predominance of common genotypes such as G1P[8], G4P[8] and [9] and only limited circulation of the emerging G12P[8] genotype. The 2015/16 surveillance period represented an unusual season, marked both by the absence of the RVA peak of infection in winter and early spring typical in Italy and other countries with temperate climate [35] and by the emergence and predominance of the G12P[8] genotype. Since RVA is not a notifiable disease in Italy, we cannot exclude the possibility that a bias in case reporting to RotaNet-Italy might have occurred. 

The number of 700 samples per year established by EuroRotaNet for Italy has been calculated as about one sample for 100,000 inhabitants, after taking into account that the Italian population is about 61,000,000 inhabitants. Children aged 0–14 years (paediatric age) are about 8,000,000 in Italy, therefore EuroRotanet recommended that sampling should reach one sample representing 12,000 inhabitants. Four of 13 regions involved in the RVA AGE surveillance were fell short of the theoretical sampling fraction value, three are very small regions in terms of paediatric population (Sardinia, Basilicata and Marche: overall 460,000 children), while Tuscany region could represent a source of bias as the population is 5,000,000 inhabitants and children aged 0-14 are about 460,000 [33]. Overall, these four regions represented a lack of 240 samples from the areas involved, which generated a bias in case reporting. In addition, we observed an oversampling in northern Italy during the 2015/16 season. However, the seasonal peak in northern Italy was present but not strongly defined, resulting in a flat season concerning sampling. For this reason the overall results possibly had a low-level of bias in case reporting.

The absence of a seasonal peak might also be linked to unusual climate, as was observed in the last decade for several other pathogens including viruses, bacteria and parasites [36-38]. In fact, the years 2015 and 2016 in Italy were characterised by unusually high temperature and humidity which stably persisted during the whole winter season [39].

The 2015/16 genotyping data revealed a decrease in the G1P[8] and G4P[8] RVA strains together with the circulation of the G12P[8] genotype in Italy. This trend was confirmed also in the 2016/17 season, where the G4P[8] strains was almost absent. The decreasing trend for the G1P[8] and G4P[8] genotypes was also confirmed by the statistical analysis, considering both the whole set of Italian data and the data collected separately from three major Italian areas. Interestingly, the frequency (6.49%) of infections with mixed genotypes detected in our study appears in line with the mean European detection rate of mixed RVA genotypes (6%). This finding represents a decreasing trend for mixed infections in Italy; previously, between 2007 and 2014, the frequency of mixed RVA infection in Italy had been assessed at 12%, the highest of the countries participating in EuroRotaNet, together with Hungary and Denmark (10%) [40].

In this study, it was not possible to define the rate of overall RVA illness because the denominators (number of negative samples, number of positive samples for other causes than RVA) were not specifically identified. Nevertheless, a regional geographical distribution of RVA genotypes was clearly defined, establishing areas where the circulation highlighted the need for accurate epidemiological studies in order to improve vaccination against RVA. The vaccination was introduced in the Italian National Immunisation Plan (PNPV 2017–2019) in January 2017, and the national coverage (20 regions) was assessed at 8.43% after the first year [41]. When considering only the 13 regions involved in the Italian surveillance, the coverage decreases to 3.98%, since the two regions not involved in the RotaNet-Italia net have the largest anti-RVA vaccination coverage: Liguria with 18.78% and Sicily with 40.80% [41]. 

Interestingly, the epidemiological findings linked to clinical data obtained in this study revealed that, older children were affected by the G12P[8] RVA genotype rather than the other genotypes (Supplement S2). This may depend on reinfection of older children with the emerging G12 genotype after its spread into the general paediatric population in Italy, resulting in a shift of the mean age of children affected [42].

The circulation and predominance of the G12P[8] RVA strains were reported throughout Italy, with a marked predominance in central Italy. In addition, all the strains shared 99–100% sequence identity with modern G12P[8] strains previously detected in Italy [29] as well as worldwide. Although the current study indicates that 49% of RVA AGE cases detected in Italy in the period from 2014 to 2017 were caused by either of the two VP7 genotypes G12 and G9, not included in any vaccine, there is a large evidence of the current vaccines’ efficacy against homotypic strains, partly heterotypic strains and even fully heterotypic strains [43-46]. Nonetheless, further investigations are desirable. Despite the documented cross-reaction between rotavirus serotypes [2,3], a recent study has highlighted the ability of this genotype to escape vaccine-induced immunity, causing symptoms in fully vaccinated children and representing a risk for the paediatric population, even in a scenario with 100% vaccination coverage [47]. 

To overcome the limitations mainly linked to the collection of samples and to the assessment of denominators, the establishment of an active institutional surveillance for AGE would be a concrete tool for the control and reduction of the disease and consequently the rate of hospitalisations. Our findings highlight the need for constant monitoring of the RVA strains circulating in Italy. The effort made by the Italian RVA surveillance programme will improve information on the epidemiology of this pathogen, supporting the evaluation of the effectiveness of rotavirus vaccines. 

## Conclusion

The findings reported in this paper highlight the emergence of the G12P[8] genotype in the Italian paediatric population. This genotype was detected for the first time in Italy in 2015, before the introduction of anti-RVA vaccination, and continued to circulate in conjunction with the improvement of vaccination. For this reason, further studies will be needed to establish the efficacy of the vaccine in a population where RVA variants other than those included in the vaccine composition circulate. Our data suggest that rotaviruses are in continuous evolution, and constant monitoring of the circulation of RVA strains is critical for an overall better knowledge of the epidemiology of this enteric pathogen in humans. The surveillance of RVA strains and the definition of their circulation pattern will prove useful for assessing the effectiveness of any potential vaccine-driven change in rotavirus strain distribution.

## Supplementary Data

176000 Supplement_S1

## Supplementary Data

69000 Supplement­_S2

## References

[1] Tate JE Burton AH Boschi-Pinto C Parashar UD World Health Organization–Coordinated Global Rotavirus Surveillance Network Global, regional, and national estimates of rotavirus mortality in children <5 years of age, 2000-2013. Clin Infect Dis. 2016;62(Suppl 2):S96-105. 10.1093/cid/civ1013 27059362 PMC11979873

[2] Estes MK Cohen J Rotavirus gene structure and function. Microbiol Rev. 1989;53(4):410-49. 2556635 10.1128/mr.53.4.410-449.1989 PMC372748

[3] Desselberger U Rotaviruses. Virus Res. 2014;190:75-96. 10.1016/j.virusres.2014.06.016 25016036

[4] Mihalov-Kovács E Gellért Á Marton S Farkas SL Fehér E Oldal M Candidate new rotavirus species in sheltered dogs, Hungary. Emerg Infect Dis. 2015;21(4):660-3. 10.3201/eid2104.141370 25811414 PMC4378476

[5] Gentsch JR Glass RI Woods P Gouvea V Gorziglia M Flores J Identification of group A rotavirus gene 4 types by polymerase chain reaction. J Clin Microbiol. 1992;30(6):1365-73. 1320625 10.1128/jcm.30.6.1365-1373.1992 PMC265294

[6] Gouvea V Glass RI Woods P Taniguchi K Clark HF Forrester B Polymerase chain reaction amplification and typing of rotavirus nucleic acid from stool specimens. J Clin Microbiol. 1990;28(2):276-82. 2155916 10.1128/jcm.28.2.276-282.1990 PMC269590

[7] Rotavirus classification working group; List of accepted genotypes Leuven. Laboratory of Viral Metagenomics. [Accessed: 2 Apr 2019]. Available from: https://rega.kuleuven.be/cev/viralmetagenomics/virus-classification/newgenotypes

[8] Iturriza-Gómara M Dallman T Bányai K Böttiger B Buesa J Diedrich S Rotavirus genotypes co-circulating in Europe between 2006 and 2009 as determined by EuroRotaNet, a pan-European collaborative strain surveillance network. Epidemiol Infect. 2011;139(6):895-909. 10.1017/S0950268810001810 20707941

[9] Matthijnssens J Van Ranst M Genotype constellation and evolution of group A rotaviruses infecting humans. Curr Opin Virol. 2012;2(4):426-33. 10.1016/j.coviro.2012.04.007 22683209

[10] Santos N Hoshino Y Global distribution of rotavirus serotypes/genotypes and its implication for the development and implementation of an effective rotavirus vaccine. Rev Med Virol. 2005;15(1):29-56. 10.1002/rmv.448 15484186

[11] Anderson EJ Weber SG Rotavirus infection in adults. Lancet Infect Dis. 2004;4(2):91-9. 10.1016/S1473-3099(04)00928-4 14871633 PMC7106507

[12] Ramig RF Genetics of the rotaviruses. Annu Rev Microbiol. 1997;51(1):225-55. 10.1146/annurev.micro.51.1.225 9343350

[13] Linhares AC Velázquez FR Pérez-Schael I Sáez-Llorens X Abate H Espinoza F Efficacy and safety of an oral live attenuated human rotavirus vaccine against rotavirus gastroenteritis during the first 2 years of life in Latin American infants: a randomised, double-blind, placebo-controlled phase III study. Lancet. 2008;371(9619):1181-9. 10.1016/S0140-6736(08)60524-3 18395579

[14] Ruiz-Palacios GM Pérez-Schael I Velázquez FR Abate H Breuer T Clemens SC Safety and efficacy of an attenuated vaccine against severe rotavirus gastroenteritis. N Engl J Med. 2006;354(1):11-22. 10.1056/NEJMoa052434 16394298

[15] Vesikari T Matson DO Dennehy P Van Damme P Santosham M Rodriguez Z Safety and efficacy of a pentavalent human-bovine (WC3) reassortant rotavirus vaccine. N Engl J Med. 2006;354(1):23-33. 10.1056/NEJMoa052664 16394299

[16] Sánchez-Uribe E Esparza-Aguilar M Parashar UD Richardson V Sustained reduction of childhood diarrhea-related mortality and hospitalizations in Mexico after rotavirus vaccine universalization. Clin Infect Dis. 2016;62(Suppl 2):S133-9. 10.1093/cid/civ1205 27059347 PMC11345715

[17] Ruggeri FM Delogu R Petouchoff T Tcheremenskaia O De Petris S Fiore L Molecular characterization of rotavirus strains from children with diarrhea in Italy, 2007-2009. J Med Virol. 2011;83(9):1657-68. 10.1002/jmv.22163 21739459

[18] Gentsch JR Laird AR Bielfelt B Griffin DD Banyai K Ramachandran M Serotype diversity and reassortment between human and animal rotavirus strains: implications for rotavirus vaccine programs. J Infect Dis. 2005;192(s1) Suppl 1;S146-59. 10.1086/431499 16088798

[19] Mita V Arigliani M Zaratti L Arigliani R Franco E Italian physicians’ opinions on rotavirus vaccine implementation. Pathogens. 2017;6(4):E56. 10.3390/pathogens6040056 29099756 PMC5750580

[20] Costantino C Restivo V Tramuto F Casuccio A Vitale F Universal rotavirus vaccination program in Sicily: Reduction in health burden and cost despite low vaccination coverage. Hum Vaccin Immunother. 2018;14(9):2297-302. 10.1080/21645515.2018.1471306 29757707 PMC6183134

[21] Kobayashi N Lintag IC Urasawa T Taniguchi K Saniel MC Urasawa S Unusual human rotavirus strains having subgroup I specificity and "long" RNA electropherotype. Arch Virol. 1989;109(1-2):11-23. 10.1007/BF01310514 2558627

[22] De Grazia S Dóró R Bonura F Marton S Cascio A Martella V Complete genome analysis of contemporary G12P[8] rotaviruses reveals heterogeneity within Wa-like genomic constellation. Infect Genet Evol. 2016;44:85-93. 10.1016/j.meegid.2016.06.039 27353490

[23] Ide T Komoto S Higo-Moriguchi K Htun KW Myint YY Myat TW Whole genomic analysis of human G12P[6] and G12P[8] rotavirus strains that have emerged in Myanmar. PLoS One. 2015;10(5):e0124965. 10.1371/journal.pone.0124965 25938434 PMC4418666

[24] Nakagomi T Do LP Agbemabiese CA Kaneko M Gauchan P Doan YH Whole-genome characterisation of G12P[6] rotavirus strains possessing two distinct genotype constellations co-circulating in Blantyre, Malawi, 2008. Arch Virol. 2017;162(1):213-26. 10.1007/s00705-016-3103-5 27718073

[25] Cilla G Montes M Gomariz M Alkorta M Iturzaeta A Perez-Yarza EG Rotavirus genotypes in children in the Basque Country (North of Spain): rapid and intense emergence of the G12[P8] genotype. Epidemiol Infect. 2013;141(4):868-74. 10.1017/S0950268812001306 22873952 PMC9151868

[26] González-Ochoa G J G Calleja-García PM Rosas-Rodríguez JA Virgen-Ortíz A Tamez-Guerra P Detection of emerging rotavirus G12P[8] in Sonora, México. Acta Virol. 2016;60(2):136-42. 10.4149/av_2016_02_136 27265462

[27] Wylie KM Weinstock GM Storch GA Emergence of rotavirus G12P[8] in St. Louis during the 2012-2013 rotavirus season. J Pediatric Infect Dis Soc. 2015;4(4):e84-9. 10.1093/jpids/piu090 26513823 PMC4681384

[28] da Silva MF Fumian TM de Assis RM Fialho AM Carvalho-Costa FA da Silva Ribeiro de Andrade J VP7 and VP8* genetic characterization of group A rotavirus genotype G12P[8]: Emergence and spreading in the Eastern Brazilian coast in 2014. J Med Virol. 2017;89(1):64-70. 10.1002/jmv.24605 27322509

[29] Delogu R Ianiro G Camilloni B Fiore L Ruggeri FM Unexpected spreading of G12P[8] rotavirus strains among young children in a small area of central Italy. J Med Virol. 2015;87(8):1292-302. 10.1002/jmv.24180 25758365

[30] Giammanco GM Bonura F DI Bernardo F Cascio A Ferrera G Dones P Introduction and prolonged circulation of G12 rotaviruses in Sicily. Epidemiol Infect. 2016;144(9):1943-50. 10.1017/S0950268815003258 26743189 PMC9150641

[31] EuroRotaNet. Rotavirus detection and typing methods. UK: EuroRotaNet; 2009. Available from: http://www.eurorota.net/docs.php

[32] Iturriza-Gómara M Kang G Gray J Rotavirus genotyping: keeping up with an evolving population of human rotaviruses. J Clin Virol. 2004;31(4):259-65. 10.1016/j.jcv.2004.04.009 15494266

[33] Italian National Institute for Statistics. Indicatori demografici. [Demographical indicators.] Rome: Instituto nazionale di Statistica. [Accessed 2 Apr 2019]. Italian. Available from: https://www.istat.it/it/archivio/indicatori+demografici

[34] Bányai K László B Duque J Steele AD Nelson EA Gentsch JR Systematic review of regional and temporal trends in global rotavirus strain diversity in the pre rotavirus vaccine era: insights for understanding the impact of rotavirus vaccination programs. Vaccine. 2012;30(Suppl 1):A122-30. 10.1016/j.vaccine.2011.09.111 22520121

[35] Wang P Goggins WB Chan EYY A time-series study of the association of rainfall, relative humidity and ambient temperature with hospitalizations for rotavirus and norovirus infection among children in Hong Kong. Sci Total Environ. 2018;643:414-22. 10.1016/j.scitotenv.2018.06.189 29940452

[36] Barril PA Fumian TM Prez VE Gil PI Martínez LC Giordano MO Rotavirus seasonality in urban sewage from Argentina: effect of meteorological variables on the viral load and the genetic diversity. Environ Res. 2015;138:409-15. 10.1016/j.envres.2015.03.004 25777068

[37] Viboud C Pakdaman K Boëlle PY Wilson ML Myers MF Valleron AJ Association of influenza epidemics with global climate variability. Eur J Epidemiol. 2004;19(11):1055-9. 10.1007/s10654-004-2450-9 15648600

[38] Wu X Lu Y Zhou S Chen L Xu B Impact of climate change on human infectious diseases: Empirical evidence and human adaptation. Environ Int. 2016;86:14-23. 10.1016/j.envint.2015.09.007 26479830

[39] Italian Ministry of Defence. Meteo aeronautica. [Aeronautic meteorology]. Rome: Ministera della Difesa. [Accessed: 2 Apr 2019]. Italian. Available from: www.meteoam.it/

[40] EuroRotaNet. Annual report 2015. Liverpool: University of Liverpool; 2016. Available from: https://docplayer.net/53024487-Eurorotanet-annual-report-2015.html

[41] Italian Ministry of Health. Vaccinazioni dell'età pediatrica. Anno 2017 (coorte 2013). [Vaccinations at paediatric age. Year 2007 (2003 cohort)]. Rome: Ministero della Salute; 2018. Available from: http://www.salute.gov.it/imgs/C_17_tavole_20_allegati_iitemAllegati_2_fileAllegati_itemFile_6_file.pdf

[42] Bishop RF Barnes GL Cipriani E Lund JS Clinical immunity after neonatal rotavirus infection. A prospective longitudinal study in young children. N Engl J Med. 1983;309(2):72-6. 10.1056/NEJM198307143090203 6304516

[43] Angel J Franco MA Greenberg HB Rotavirus immune responses and correlates of protection. Curr Opin Virol. 2012;2(4):419-25. 10.1016/j.coviro.2012.05.003 22677178 PMC3422408

[44] Leshem E Lopman B Glass R Gentsch J Bányai K Parashar U Distribution of rotavirus strains and strain-specific effectiveness of the rotavirus vaccine after its introduction: a systematic review and meta-analysis. Lancet Infect Dis. 2014;14(9):847-56. 10.1016/S1473-3099(14)70832-1 25082561

[45] Payne DC Boom JA Staat MA Edwards KM Szilagyi PG Klein EJ Effectiveness of pentavalent and monovalent rotavirus vaccines in concurrent use among US children <5 years of age, 2009-2011. Clin Infect Dis. 2013;57(1):13-20. 10.1093/cid/cit164 23487388 PMC4618548

[46] Steele AD Neuzil KM Cunliffe NA Madhi SA Bos P Ngwira B Human rotavirus vaccine Rotarix™ provides protection against diverse circulating rotavirus strains in African infants: a randomized controlled trial. BMC Infect Dis. 2012;12(1):213. 10.1186/1471-2334-12-213 22974466 PMC3462149

[47] Ogden KM Tan Y Akopov A Stewart LS McHenry R Fonnesbeck CJ Multiple introductions and antigenic mismatch with vaccines may contribute to increased predominance of G12P[8] rotaviruses in the United States. J Virol. 2018;93(1):e01476-18. 10.1128/JVI.01476-18 30333170 PMC6288334

