# Supplementary material for: Group A rotavirus surveillance before vaccine introduction in Italy, September 2014 to August 2017
Source: Euro Surveill. 2019 Apr 11;24(15):1800418. doi: 10.2807/1560-7917.ES.2019.24.15.1800418 (PMC6470368; doi:10.2807/1560-7917.ES.2019.24.15.1800418)
Supplement: Supplement_S1 [file 1800418_IANIRO_SupplementS1.pdf]

Supplement 1.

RotaNet-Italy surveillance questionnaire provided to all hospitals involved in the surveillance net, and sent back to ISS in association to each fecal sample.

This supplementary material is hosted by Eurosurveillance as supporting information alongside the article "Group A rotavirus surveillance before vaccine introduction in Italy, 2014 to 2017" on behalf of the authors who remain responsible for the accuracy and appropriateness of the content. The same standards for ethics, copyright, attributions and permissions as for the article apply. Eurosurveillance is not responsible for the maintenance of any links or email addresses provided therein.

Date ____/___/____

Rotavirus Surveillance

Country _________________ City_____________________ Hospital code ________________

Sample code ____ Sex _______________________

City of birth____ Date of birth ___/___/___

City of residence ____________________________ Living setting Urban ☐ Rural ☐

Ward of admission__________________ Hospital address______________________________

Cause of admission __________________________________ Date of admission ___/____/____

Nosocomial infection Yes ☐ No ☐

Symptoms:

Fever (>38°C): Yes ☐ No ☐

Diarrhea: > 3 discharge/day ☐ < 3 discharge/day ☐ No ☐

Vomit: Yes ☐ No ☐

Abdominal pain: Yes ☐ No ☐

Rotavirus vaccination Yes ☐ No ☐ Dose_________ Vaccine used__________

Other symptoms: ____

Other recent vaccinations: ____

Other cases (house or school):____

Date of collection of feces: ___/____/____

Name of the physician ____

Phone ___________________ fax __________________ E-mail ___________________________
