# Supplementary material for: Group A rotavirus surveillance before vaccine introduction in Italy, September 2014 to August 2017
Source: Euro Surveill. 2019 Apr 11;24(15):1800418. doi: 10.2807/1560-7917.ES.2019.24.15.1800418 (PMC6470368; doi:10.2807/1560-7917.ES.2019.24.15.1800418)
Supplement: Supplement­_S2 [file 1800418_IANIRO_SupplementS2.pdf]

Supplement 2.

Statistical analysis of the RVA infections considering the age (months) of patients affected. Values with a statistical significance are highlighted in red.

This supplementary material is hosted by Eurosurveillance as supporting information alongside the article “Group A rotavirus surveillance before vaccine introduction in Italy, 2014 to 2017” on behalf of the authors who remain responsible for the accuracy and appropriateness of the content. The same standards for ethics, copyright, attributions and permissions as for the article apply. Eurosurveillance is not responsible for the maintenance of any links or email addresses provided therein

| Months | G12P[8] | G1P[8] | G2P[4] | G3P[8] | G4P[8] | G9P[8] | Mixed | Uncommon | All except the group |
|---|---|---|---|---|---|---|---|---|---|
| G12P[8] | x | 0.2491 | 0.5964 | 0.1679 | 0.1771 | 0.0452 | 0.9936 | 0.8417 | 0.0452 |
| G1P[8] | | x | 0.1602 | 0.2442 | 0.8304 | 0.4342 | 0.3608 | 0.9933 | 0.6564 |
| G2P[4] | | | x | 0.0118 | 0.0207 | 0.0055 | 0.5749 | 0.6023 | 0.1473 |
| G3P[8] | | | | x | 0.0499 | 0.1288 | 0.0907 | 0.1301 | 0.1629 |
| G4P[8] | | | | | x | 0.4635 | 0.1623 | 0.9579 | 0.5077 |
| G9P[8] | | | | | | x | 0.0526 | 0.8435 | 0.1134 |
| Mixed | | | | | | | x | 0.805 | 0.3504 |
| Uncommon | | | | | | | | x | 0.9487 |
| All except the group | | | | | | | | | x |
